# mRNA Vaccines Encoding the HA Protein of Influenza A H1N1 Virus Delivered by Cationic Lipid Nanoparticles Induce Protective Immune Responses in Mice

**DOI:** 10.3390/vaccines8010123

**Published:** 2020-03-10

**Authors:** Xinyu Zhuang, Yanxin Qi, Maopeng Wang, Ning Yu, Fulong Nan, He Zhang, Mingyao Tian, Chang Li, Huijun Lu, Ningyi Jin

**Affiliations:** 1 Key Laboratory of Jilin Province for Zoonosis Prevention and Control, Institute of Military Veterinary Medicine, Academy of Military Medical Sciences, Academy of Military Sciences, Changchun, Jilin 130000, China; xinyuzhuang367@163.com (X.Z.); hezhangvs@126.com (H.Z.); klwklw@126.com (M.T.); lichang78@163.com (C.L.); huijun_lu@126.com (H.L.); 2 State Key Laboratory of Polymer Physics and Chemistry, Changchun Institute of Applied Chemistry, Chinese Academy of Sciences, Changchu 130022, China; yxqi@ciac.ac.cn; 3 Institute of Virology, Wenzhou University, Wenzhou, Zhejiang 325000, China; wangmaopenga@126.com; 4 Department of Veterinary Medicine, College of Agriculture, Yanbian University, Yanji, Jilin 133000, China; yn8597@126.com; 5 College of Veterinary Medicine, Jilin University, Changchun, Jilin 130000, China; nflcom@163.com

**Keywords:** mRNA vaccine, cationic lipid nanoparticles, mannose, influenza A H1N1 virus, intranasal administration

## Abstract

The design of the mRNA vaccine involves the selection of in vitro transcription (IVT) systems and nonviral delivery vectors. This study aimed to verify the effect of 5’ and 3’ untranslated region (UTR) sequences on the translation efficiency of mRNA. Three modes of IVT-mRNA systems (IVT-mRNA-n1/n2/n3) with diverse UTRs were constructed, and EGFP (enhanced green fluorescent protein) and HA (hemagglutinin) gene of H3N2 influenza virus were introduced into each of them. The results showed that the mode of 5’ and 3’ UTRs originating from human β-globulin was better than the mode of UTRs from human α-globulin, and the n3 mode was the best. mEGFP-n3, mH3HA-n3, and mLuciferease-n3 were prepared to compare the effect of cationic lipid nanoparticle (LNP) with that of mannose-conjugated LNP (LNP-Man) on the efficiency of gene delivery. The results showed that the effect of LNP-Man was better than that of LNP both in vitro and in vivo. Choosing appropriate ligands might help in vaccine design. After selecting the IVT-mRNA-n3 system and delivery vectors, mRNA vaccines were constructed against the H1N1 influenza virus, and C57BL/6 mice were immunized through intranasal administration. The results showed that mRNA vaccines could elicit both humoral and cellular immune responses and completely protect mice from the tenfold LD_50_ H1N1 influenza virus challenge.

## 1. Introduction

Influenza viruses belong to the family of Orthomyxoviridae, with negative-sence, single-stranded, and segmental RNA genomes. Influenza A and B viruses cause threats to human health they can give rise to mild-to-severe respiratory infections. According to the statistics of the World Health Organization, seasonal influenza viruses (A/H1N1/H3N2), as well as influenza B viruses (B/Yamagata/Victoria), account for nearly 3–5 million critical cases and 0.29–0.65 million deaths worldwide each year [1]. Vaccination is the best protection measure to prevent influenza. However, seasonal influenza viruses can evolve constantly through antigen drift and antigen shift. These punctuated antigenic changes, especially in HA (the main target of protective antibodies), lead to evasion of the immunity elicited by previous infection or vaccination [2,3]. Therefore, vaccine strains need to be renewed annually [4]. With the increase in globalization and the emergence of new pathogens, a new vaccine platform is urgently needed to solve the time lag between the emergence of pathogens and vaccine licensing [5].

Currently, three kinds of vaccines are licensed in various countries: inactivated, live attenuated, and recombinant HA vaccines. In addition, DNA/RNA-based vaccines, recombinant protein vaccines, virus-like particles, and viral vector-based adjuvanted vaccine strategies are undergoing clinical trials. Each vaccine has its own advantages and disadvantages. Compared with other types of vaccines, mRNA vaccines have many advantages, such as no anti-vector immunity and no egg-adaptive mutations. During chicken embryo culture, certain viruses can produce mutations that adapt to eggs, thereby altering the antigenicity of virus’s surface proteins. The DNA vaccine must pass through the cell membrane and nuclear membrane before transcription, while the mRNA vaccine can be translated immediately after the mRNA enters the cytoplasm. Furthermore, the protein expression of the mRNA vaccine is transient, avoiding T-cell depletion due to continuous antigen exposure [6].

At present, mRNA-based vaccines have been widely studied in both infectious and noninfectious diseases, some of which are undergoing clinical trials [7,8,9,10,11,12,13]. The two main types of RNA vaccines used against influenza viruses are self-amplified mRNA (SAM) vaccine and nonreplicating mRNA vaccine. A pioneering work showed that intramuscular injection of SAM encoding the HA gene in A/California/07/2009, combined with oil-in-water nanoemulsions, could protect mice and ferrets from homologous and heterogeneous influenza viruses [4,14]. Barr and colleagues provided evidence that a single intradermal immunization with a 10-µg mRNA vaccine encoding the nucleoside-modified HA gene of H10N8 or H7N9 led to the production of HA antibodies in mice for more than a year. Phase I clinical trials (NCT03076385) using this platform were launched due to promising preclinical results [14,15].

Many factors can affect the efficacy of mRNA vaccines, including optimization of target genes, incorporation of UTRs, selection of delivery vectors, modification of ligands, and addition of vaccine adjuvants. 5ʹ- and 3ʹ-UTRs containing regulatory sequence elements that have been identified to modulate the translation and stability of endogenous mRNA [16,17]. The sequences in 5’UTR can inhibit 5’-exonucleolytic degradation and the sequences in 3’UTR can repress deadenylation of mRNA. In addition, various regions of cellular and viral 5ʹ- and 3ʹ-UTRs enhance the stability and translational efficiency of mRNA [18].

As nonviral delivery systems, nanoparticles provide a variety of platforms for the expression of exogenous mRNA in vivo [19]. Cationic lipid nanoparticles are increasingly regarded as one of the most promising mRNA delivery systems because of their good biocompatibility and easy large-scale production. Cationic lipid/mRNA nanoparticles can protect mRNA from degradation by nucleases and deliver it into cells by electrostatic adsorption and fusion with the cell membrane [20]. Lipophilic molecules equipped with ligands can specifically recognize receptors on a cell and improve delivery efficiency [21]. For example, mannose receptors are expressed on antigen-presenting cells, especially macrophages and dendritic cells (DCs), and are the candidate target for vaccines to introduce genes encoding various antigens [22,23,24].

In this study, three modes of in vitro transcription mRNA systems (IVT-mRNA-n1/n2/n3) were constructed. The efficiency of foreign gene expression was compared among these modes through in vitro experiments, and IVT-mRNA-n3 with the highest translation efficiency of mRNA was selected for follow-up experiments. Cationic lipid nanoparticles (LNPs) and mannose-conjugated LNP (LNP-Man) were prepared to encapsulate mRNA. The effect of LNP-Man was better than that of LNP both in vitro and in vivo. Finally, the mRNA vaccine encoding the HA gene of H1N1 influenza A virus was encapsulated separately with two kinds of LNPs, and the immunogenicity and protective effect against the tenfold LD_50_ virus challenge in mice were evaluated.

## 2. Materials and Methods

### 2.1. Cells, Mice and Influenza Virus

A549 (human non-small cell lung cancer cells), BHK-21 (baby hamster Syrian kidney cells), MDCK (Madin-Darby canine kidney cells), HeLa (Henrietta Lacks cells), HEK-293 (human embryo kidney cells), BEAS-2B (human bronchial epithelial cells), and NCI-H226 (human lung squamous carcinoma cells) were all preserved by our laboratory—Department of Virology, Institute of Military Veterinary Medicine, Academy of Military Medical Sciences, Academy of Military Sciences, Changchun, China. Specific pathogen-free female C57BL/6 mice were purchased from Beijing Vital River Laboratory Animal Technology Co., Ltd., Beijing, China.

A/Jilin/JYT-01/2018(H1N1) virus was also preserved by our laboratory. All experiments involving live viruses were performed in biosafety level 3 facilities.

### 2.2. Construction of the Plasmids Expressing EGFP and H3N2-HA Protein

The pGEM-3Zf (+) vector (Promega, Madison, WI, USA) is intended for the highly efficient synthesis of RNA in vitro. Three modes of IVT-mRNA-systems with diverse untranslated regions (UTRs) were subcloned and named as IVT-mRNA-n1/n2/n3 [12,17]. The sequences of 5’ and 3’ UTRs were derived from human α-globin or β-globin. To verify the effect of 5’ and 3’ UTR sequences on the translation efficiency of mRNA, the H3N2-HA genes cloned from A/swine/Guangdong/1/2003 (H3N2) virus and enhanced green fluorescent protein (EGFP) were introduced into each of these sequences. The concentration and purity of recombinant plasmids were determined using Nanodrop 2000 (Thermo Fisher Scientific, Waltham, MA, USA).

### 2.3. In Vitro mRNA Transcription (IVT-mRNA) 

Plasmids linearized with *Xho*I (New England Biolabs, Beverly, MA, USA) were used as DNA templates for in vitro transcription reaction, according to the protocol provided with a T7-FlashScribe Transcription Kit (CELLSCRIPT, Madison, WI, USA). 1-Methylpseudouridine-5′-triphosphate (TriLink, NorthPark, CA, USA) was used instead of uridine triphosphate (UTP). Then, the mRNA was capped using a ScriptCap Cap 1 Capping System (CELLSCRIPT, Madison, WI, USA) and purified using a MEGAclear Transcription Clean-Up Kit (Thermo Fisher Scientific, Waltham, MA, USA) [11]. The concentration and purity of capped mRNA were determined by absorbance at 260 nm using the Nanodrop 2000 spectrophtometer (Thermo Fisher Scientific, Waltham, MA, USA).

### 2.4. Transfection of Cap-mRNA into A549 Cells

The delivery of Cap-mRNA into cells requires the assistance of transfection reagents. In this part of the experiment, lipofectamine 3000 (Invitrogen, Carlsbad, CA, USA) was used as the transfection reagent. Briefly, A549 cells were seeded at a density of 1 × 10^5^ cells/well on 12-well plates with F12 medium (Hyclone, South Logan, UT, USA) supplemented with 10% fetal bovine serum (Gibco, Grand Island, NY, USA) and incubated at 37 °C in the presence of 5% CO_2_ until the confluence reached 80%. The mixed lipofectamine 3000/Cap-mRNA at a volume/mass ratio of 3 μL/1 μg was added into wells, following the manufacturer’s instructions. 

### 2.5. Flow Cytometry

The percentage of EGFP-positive cells in each group was detected by flow cytometry (BD Biosciences, San Jose, CA, USA). After 12 h, the cells transfected with Cap-mEGFP-n1/n2/n3 were digested with trypsin, washed twice with phosphate-buffered saline (PBS), and finally, resuspended in 1 mL of PBS. Ten thousand cells were collected from each group, and the green fluorescence signal was captured through a fluorescein isothiocyanate (FITC) channel.

### 2.6. Western Blot Analysis

After 12 h, the cells transfected with Cap-mH3HA-n1/n2/n3 were verified by Western blot analysis. The cells were collected, and radio immunoprecipitation assay (RIPA) lysate and phenylmethanesulfonyl fluoride were used to lyse the cells. The total protein of the cells was extracted, and a bicinchoninic acid assay kit (Biyuntian Biotechnology Co. Ltd., Shanghai, China) was used to measure the protein concentration. The same amount of protein was taken for SDS-polyacrylamide gel electrophoresis separation, transferred on to the nitrocellulose membrane (GE Healthcare, Little Chalfont, Buckinghamshire, UK), blocked with 5% skimmed milk, mixed with influenza A virus HA rabbit Mab (1:2000) (Sino Biological, Beijing, China) at room temperature for 2 h, and washed thrice with TBST for 8 min each. It was then incubated with the secondary antibody for 1 h at room temperature and washed thrice with TBST for 8 min each. Further, the blots were developed using Clarity Western ECL Substrate (Thermo Fisher Scientific, Waltham, MA, USA) and visualized using Amersham Imager 600 (GE Healthcare, Little Chalfont, Buckinghamshire, UK). The gray value of the strips were analyzed using ImageJ software, and the bar chart was drawn using GraphPad Prism 8.0.

### 2.7. Formulation of LNPs/mRNA

LNP comprised 1, 2-dioleoyl-3-trimethylammonium-propane (DOTAP), 1, 2-dioleoyl-sn-glycero-3-phosphoethanolamine (DOPE), and 1, 2-distearoyl-sn-glycero-3-phosphoethanolamine-N-(methoxy(polyethylene glycol)-2000) (DSPE-mPEG2000) (50:50:1 mol/mol). LNP-Man contained DOTAP, DOPE, and DSPE-PEG-Mannose (50:50:1 mol/mol). DOTAP and DOPE were purchased from A.V.T. Pharmaceutical Co., Ltd. (Shanghai, China). DSPE-PEG-Mannose was purchased from Xi’an ruixi Biological Technology Co., Ltd. (Shanxi, China). Both were produced by the thin-film hydration method [25,26,27]. Briefly, all the materials were dissolved in chloroform at the indicated molar ratios and then evaporated using a rotatory evaporator for 30 min. The obtained dry film was hydrated with 10mM HEPES buffer and dispersed in a water bath supersonic device for 30 min. Finally, the solutions were filtered through a 0.22-μm sterile filter. The size and zeta potential were measured using Malvern Zetasizer Nano ZS90 (Malvern Panalytical, Westborough, MA, UK).

LNPs/mRNA were prepared at the indicated N:P molar ratio (N, nitrogen on DOTAP; P, phosphate on mRNA) by adding mRNA to LNPs and mixed with mild vortexing for 1 min at room temperature, followed by an incubation time of 30 min. A mean molar mass of 330 Da per nucleotide was assumed for calculating the molar ratio between DOTAP and RNA.

The gel retardation assay was conducted to investigate the packaging efficiency of LNPs/mRNA. The LNPs/mRNA complexes were prepared at N/P molar ratios of 16:1, 14:1, 12:1, 10:1, 8:1, and 6:1. Then, the samples mixed with 6× loading buffer (TaKaRa, Tokyo, Japan) were loaded on a 1.2% agarose gel. After running for about 30 min at 80 V, pictures were acquired using an imager (ProteinSimple, Silicon Valley, CA, USA).

### 2.8. Cytotoxicity Assay

The cytotoxicity of LNP and LNP-Man were separately measured using a Cell Counting Kit-8 (CCK-8) according to the instructions. The LNP or LNP-Man was prepared at the indicated dosages and then added to A549 cells. After 24-h incubation, 10 μL of CCK-8 buffer was added, followed by reaction at 37 °C for 4 h. Following this, the absorbance was determined at 450 nm. The cells not exposed to LNP or LNP-Man were used as control, and the survival rate was 100%.

### 2.9. In Vitro Maturation of Bone Marrow–Derived Cells

Bone marrow–derived cells (BMDCs) were produced as described in a previous study [28]. Briefly, bone marrow progenitor cells were acquired from the femurs of 6- to 8-week-old C57BL/6 female mice. The cell suspension was filtered through a cell strainer and centrifuged at 1500 rpm for 10 min. Red blood cells were lysed using ammonium-chloride-potassium lysing buffer. The cell pellets were resuspended in RPMI complete medium (Hyclone, South Logan, UT, USA) containing 20 ng/mL mouse granulocyte/macrophage colony-stimulating factor (mGM-CSF) and 20 ng/mL mouse interleukin-4 (mIL-4) (R&D Systems, Minneapolis, MN, USA) and cultivated in six-well plates. The medium was carefully replaced with a fresh complete medium every 2 days. The semi-adherent DCs were collected on the eighth day. 

A PE-anti-mouse MHC-II antibody was used to detect the purity of BMDCs. When the percentage of MHC-II positive cells was higher than 65%, BMDCs could be used for further experiments. The cells were plated on 12-well plates at a density of 2 × 10^5^ cells/well to determine the stimulation effect of mH3HA, LNP, LNP-Man, and LNPs/mH3HA on the maturation of DCs. LNPs/mH3HA (N/P = 10:1 mol/mol) containing 3 μg mH3HA were added to each well. After 48 h incubation, the cells were harvested and stained simultaneously with an FITC-labeled CD11c antibody and a PE-labeled CD80 antibody (or PE-labeled CD86 antibody) (eBioscience, San Diego, CA, USA). The cells stimulated with LPS (1 μg/1 × 10^5^ cells) were used as a positive control. The results were analyzed using a flow cytometer.

### 2.10. In Vivo Bioluminescence Imaging

LNPs/mRNA encoding firefly luciferase (Luc) was inoculated into C57BL/6 mice through intranasal (i.n.) and intramuscular (i.m.) administration (only the right leg). The dosage of mLuc was 12 μg/mouse. After 6-h inoculation, the mice were anesthetized and injected with 100 mg/kg body weight VivoGlo Luciferin (Promega, Madison, WI, USA) through intraperitoneal administration. The mice in the intranasal administration group were euthanized 10 min after injecting VivoGlo Luciferin, and the lungs were removed and photographed. Images were acquired using IVIS Lumina S5 (Perkin-Elmer, Waltham, MA, USA). Bioluminescence intensity from the region of interest was quantified using Living Image software.

### 2.11. Indirect Immunofluorescence Assay

Briefly, A549 cells were seeded at a density of 1 × 10^5^ cells/well on 12-well plates. The mixed lipofectamine 3000/Cap-mH1HA-n3 was added at a volume/mass ratio of 3 μL/1 μg to wells following the manufacturer’s protocols. The cells were fixed with 4% paraformaldehyde for 10 min and blocked with 2% BSA (bovine serum albumin) and 0.1% Triton X-100 in PBS for 30 min at room temperature 48 h after transfection. After washing with PBS twice, the cells were incubated with an influenza A virus HA antibody (Rabbit Mab; Sino Biological, Beijing, China) (1:500) for 1 h at 37 °C. FITC-conjugated goat anti-rabbit immunoglobulin G (IgG) antibodies (1:500) diluted in PBS were added and incubated for 1 h at room temperature. Finally, the cells were incubated with 4’,6-diamidino-2-phenylindole (DAPI) (0.5 μg/mL) for 10 min at room temperature. Fluorescence images were scanned using an Olympus microscope (Olympus, Tokyo, Japan).

### 2.12. Mouse Immunization and Infection

Female C57BL/6 mice, aged 6–8 weeks, were randomly divided into five groups (12 mice/group). The mice in the vaccine groups were intranasally inoculated with LNPs/mH1HA and LNP-Man/mH1HA (mH1HA = 12 μg/mouse) in weeks 0 and 3, respectively. For the negative control groups, the mice received the same volume of 0.9% NaCl solution, LNP, or LNP-Man at the same time point.

Two weeks after the boost immunization, three mice were randomly selected from each group to isolate spleen lymphocytes and evaluate the level of the vaccine-induced cellular immune response in mice. In the first, third, and fifth weeks after the first immunization, blood was collected from the orbital vein using a blood collection needle, and the collected blood was centrifuged at 3000 rpm to isolate serum (10 min at 4 °C). The serum stored in aliquots at –80 °C for subsequent ELISA (enzyme linked immunosorbent) and HAI (hemagglutination inhibition) assays that were used to evaluate the level of vaccine-induced humoral immune response.

Nine mice from each group were challenged intranasally with 10 × LD_50_ (median lethal dose) A/Jilin/JYT-01/2018(H1N1) influenza virus. The lungs from three mice of each group were removed to prepare pathological HE (hematoxylin-and-eosin-stained) sections on day 5 after infection. Six mice from each group were monitored daily for body weights and survival during 2 weeks after challenge.

#### 2.12.1. Hemagglutination Inhibition Assay

Hemagglutination inhibition (HI) assay was performed according to standard procedures [4]. First, the receptor-destroying enzyme (Denka) was added according to the instructions to inactivate nonspecific inhibitors in the serum samples. Then, the treated sera were serially diluted twofold in 96-well plates and incubated with an equal volume of inactivated whole virus for 30 min at room temperature. Finally, 0.5% (vol/vol) cock red blood cells were added, mixed, and incubated for 30 min. The results were determined by visual inspection. The HI titer was defined as the reciprocal of serum dilution at which the last complete HI occurred.

#### 2.12.2. ELISA Measurement of Antibody and Cytokine Levels in the Serum

After the initial immunization, the sera were obtained in weeks 1, 3, and 5. The total levels of IgG, IgG1, and IgG2a were assessed using ELISA kits (Genway Biotech, San Diego, CA, USA), and commercial mice cytokine ELISA kits were used to analyze the concentration of IL-4 and IFN-γ (interferon-γ) (eBioscience, San Diego, CA, USA) following the manufacturer’s protocol.

#### 2.12.3. Lung Influenza Viral Load

The right lungs of mice were collected in PBS and homogenized using a tissuelyser machine (Shanghai Jingxin Industrial Development CO., Ltd, Shanghai, China). Total RNA was extracted using virus RNA isolation kit (Sangon Biotech Co., Ltd, Shanghai, China). cDNAs were generated using the One-Step RT-PCR kit (TIANGEN Biotech Co., LTD., Shanghai, China). The cDNA served as a template for the amplification of the influenza M1 gene and eukaryotic HPRT1 housekeeping gene by real-time PCR. Quantitative RT-PCR was performed in triplicate for each cDNA sample using the Taq-Man Universal Master Mix with UNG (Thermo Fisher Scientific, Waltham, MA, USA). The primers and probe used to amplify the influenza M1 gene were: forward primer: 5-AAGACCAATCCTGTCACCTC TGA-3, reverse primer: 5-CAAGCGTCTACGCTGCAGTCC-3, and probe: 5-((6FAM) TTTGTG TTCACGCTCACCGT (TAM)). The thermal cycling program was as follows: 50°C, 2 min; 95°C, 10 min; 40 cycles of 15 s at 95°C; and 60°C, 1 min. RT-PCR was analyzed using the 7500 Real-Time PCR System (Thermo Fisher Scientific, Waltham, MA, USA). The copy numbers of the virus genes were calculated using the 2^-ΔΔCt^ method and were indicated as the fold change relative to noninfected lungs. M1 mRNA expression was normalized to levels of mouse HPRT1 mRNA expressed in the corresponding sample.

### 2.13. Laboratory Facilities and Ethics Statement

All C57BL/6 mice were treated in accordance with the provisions of the Welfare and Ethics of Laboratory Animals of China, and protocols were approved by the Animal Welfare and Ethics Committee of the Institute of Military Veterinary Medicine, Academy of Military Medical Sciences, Academy of Military Sciences (IACUC of AMMS-13-2016-006).

## 3. Results

### 3.1. Construction and Screening of IVT-mRNA Systems

**Figure 1:**
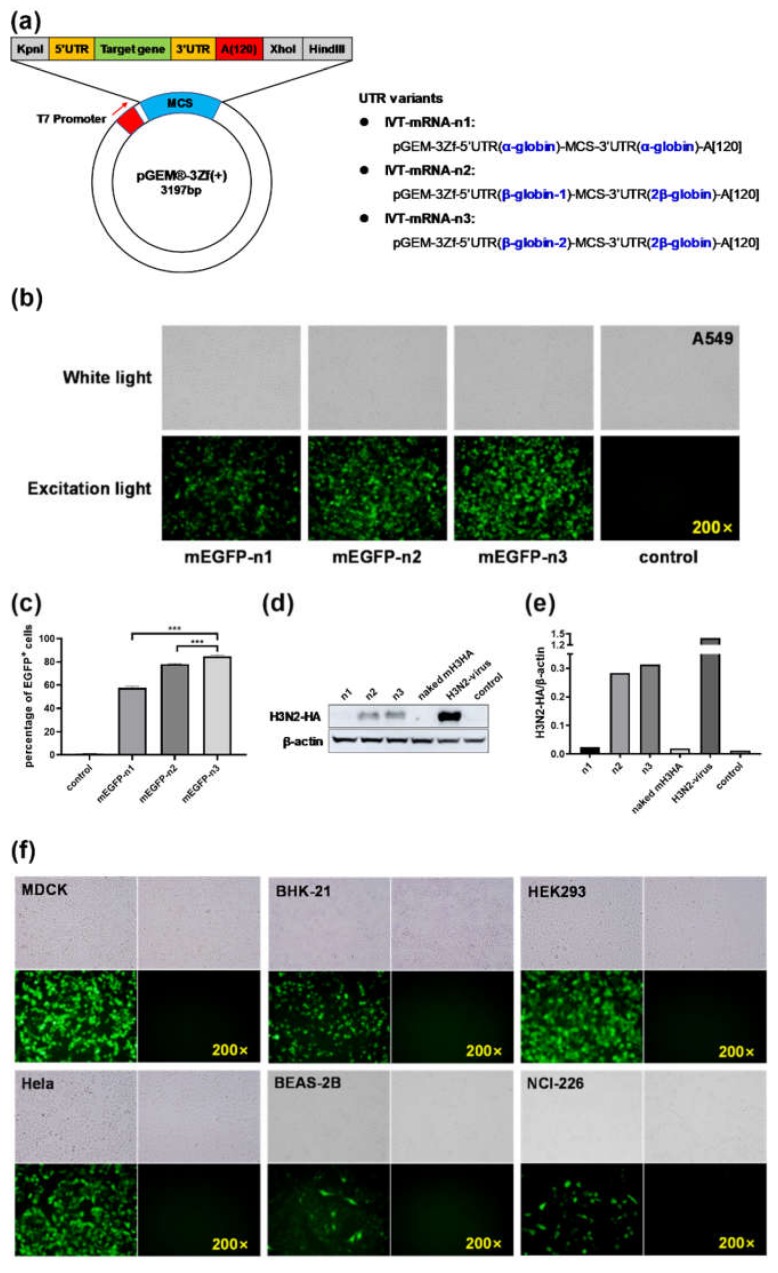
(**a**) Pattern diagram of the three modes of in vitro transcription systems. A549 cells transfected with enhanced green fluorescent protein (mEGFP)-n1/n2/n3 were observed 12 h after transfection under a fluorescence microscope (**b**), and the positive rates were detected by flow cytometry (**c**). Data are shown as means ± SDs (standard deviation) and analyzed using one-way ANOVA (analysis of variance) (*n* = 3, mEGFP-n3 vs. mEGFP-n1, *** *p* < 0.0001; mEGFP-n3 vs. mEGFP-n2, *** *p* < 0.0001). (**d,e**) Western blot analysis. A549 cells were harvested 12 h after transfection. The H3N2-HA protein was detected using rabbit anti-influenza A virus HA Mab (Sino Biological, Beijing, China). The gray value of the strips was analyzed using ImageJ software, and the bar chart was drawn using GraphPad Prism 8.0. (**f**) Fluorescence microscope images of various cell lines transfected with mEGFP-n3 after 12 h.

Three modes of IVT-mRNA-systems were named as IVT-mRNA-n1/n2/n3 (Figure 1a) and their sequences were listed in Table S1. Recombinant plasmids encoding EGFP (720 bp) or H3N2-HA (1701 bp) genes were acquired and verified by *Xho*I digestion and sequencing (Figure S1a,b). These results were consistent with the predicted length after adding UTRs and poly(A) to the vector. Fluorescence microscope images illustrated that all the three IVT-mRNA systems could express EGFP successfully (Figure 1b). The percentage of EGFP-positive cells was significantly higher in the mEGFP-n3 group than in the other two groups (Figure 1c and Figure S2a–d). The results of Western blot analysis showed that HA proteins measuring about 75 kDa could be successfully expressed in A549 cells, and the protein expression of the mH3HA-n3 group was the highest (Figure 1d,e). BHK-21, MDCK, HeLa, HEK-293, BEAS-2B, and NCI-H226 cells were transfected with mEGFP-n3 to verify the effect of the IVT-mRNA-n3 system on other cell lines. Fluorescence microscope images showed that EGFP could be well expressed in each cell line (Figure 1f). Based on the results, IVT-mRNA-n3 was selected as the final IVT-mRNA system for the follow-up experiments.

### 3.2. Characterization of LNPs and LNPs/mRNA

**Figure 2:**
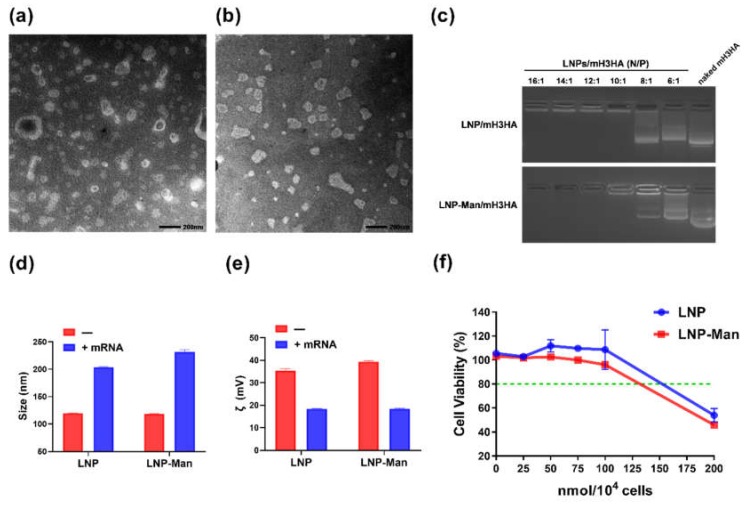
Characterization of lipid nanoparticles (LNPs) and LNPs/mRNA. (**a,b**) TEM images of LNP and LNP-Man. (**c**) Gel retardation assay. LNPs/mRNA were run in the 1.2% nuclease-free agarose gel. LNPs/mRNA complexes were prepared at different N/P molar ratios. Naked mRNA was used as the negative control without any complexation. (**d**) Size and (**e**) zeta potential of LNPs and LNPs/mH3HA (N/P = 10:1). (**f**) Cytotoxicity of LNP and LNP-Man was tested on A549 cells. Untreated cells were defined as 100% viability cells. Data are shown as means ± SDs (*n* = 3).

Transmission electron microscope (TEM) images illustrated that LNP (Figure 2a) and LNP-Man (Figure 2b) were spherical in shape. The gel retardation assay (Figure 2c) showed that the migration of mH3HA could be completely retarded using the N/P ratio of LNPs/mH3HA higher than 10:1, indicating that LNPs had a good encapsulation efficiency. The size and zeta potential of LNPs and LNPs/mH3HA (N/P = 10:1) were measured, respectively (Figure 2d,e and Figure S3a–d). LNP and LNP-Man comprised DOTAP, which is a cationic lipid. A zeta potential greater than zero indicated that the surface of the material was positively charged (the amount of positive charge was much greater than that of negative charge). On the contrary, zeta potential less than zero indicated that mRNA was negatively charged. The results are summarized in Tables S2 and S3. The findings revealed that LNPs could combine with mRNA through electrostatic interaction, resulting in an increase in LNP particle size and a decrease in zeta potential. Figure 2f shows that when the molar of N (nitrogen on DOTAP) was less than 100 nmol/10^4^ cells, the LNP and LNP-Man had low toxicity, and the cell survival rates were higher than 80%. When the dosages of LNPs reached 200 nmol/10^4^ cells, both formulations induced nearly 50% of cell death, which hindered their application in cell experiments. Therefore, the dosage of LNPs used in the follow-up in vitro cell experiments was 100 nmol/10^4^ cells.

### 3.3. Functional Verification of LNPs/mRNA

**Figure 3:**
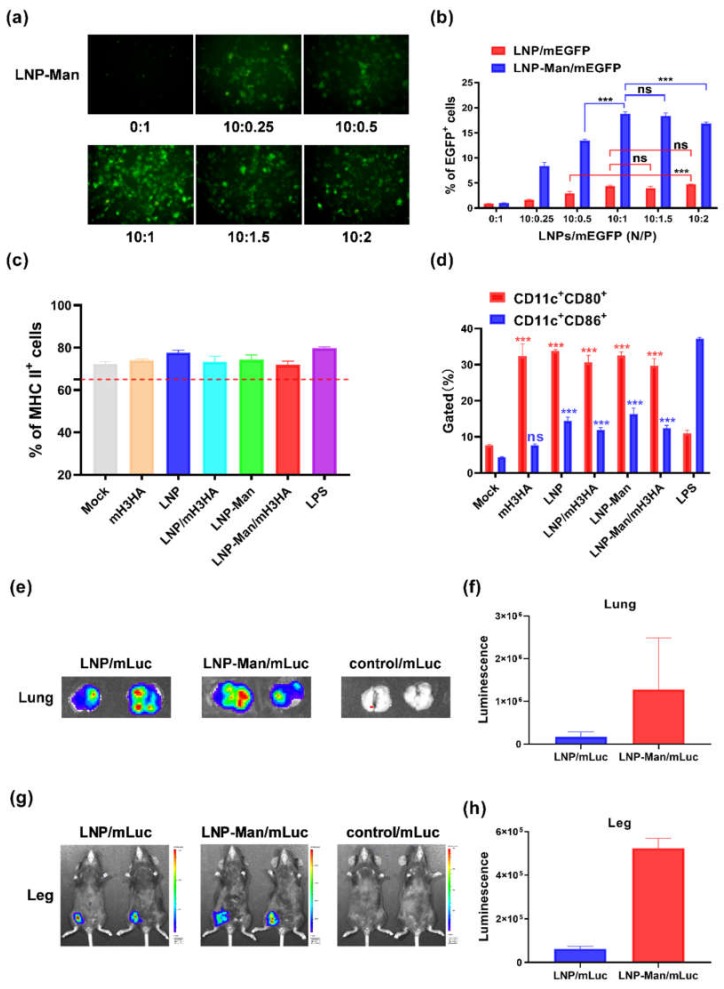
Functional verification of LNPs/mRNA. (**a**) Fluorescence microscope imaging. A549 cells transfected with LNP-Man/mEGFP at indicated N/P molar ratios were observed under a fluorescence microscope 12 h after transfection. (**b**) The EGFP positivity rates of cells transfected with LNPs/mEGFP at indicated N/P molar ratios were detected by flow cytometry. Data are shown as means ± SDs and were analyzed by two-way ANOVA (*n* = 3, ns *p* > 0.05; *** *p* < 0.0001). (**c**,**d**) Flow cytometric analysis of the dendritic cells’ (DC) maturation levels. Data are shown as means ± SDs and were analyzed by two-way ANOVA. (*n* = 3, ns *p* > 0.05; ***p < 0.0001; compared with Mock). (**e**,**f**) In vivo imaging. Images of lungs were acquired using an IVIS Lumina S5, and bioluminescence intensity from the region of interest was quantified using Living Image software. (**g,h**) In vivo imaging. Images of legs and the bioluminescence intensity.

Fluorescence microscope images (Figure 3a) showed that the EGFP was expressed successfully. However, no green fluorescence was observed in the naked mEGFP group (0:1), indicating that LNP-Man could protect mEGFP from degradation and deliver it into A549 cells. The ability of LNP and LNP-Man to deliver mEGFP into cells was also determined by flow cytometry (Figure 3b). In these experiments, the optimal N/P molar ratio of LNPs/mEGFP was 10:1, exhibiting the highest transfection efficiency. However, no significant difference in transfection efficiency was found with the increasing molar ratio of mEGFP, which might be related to the entrapment efficiency of LNPs. A part of mEGFP could not be wrapped into LNPs and delivered into cells. Therefore, the percentage of EGFP^+^ cells was no longer increased.

Dendritic cells (DCs) play a key role in the immune system, linking innate and adaptive immune responses. The maturity of DC is a key factor to be considered in vaccine design and effectiveness evaluation. The proportion of MHC-II- and CD11c-positive cells indicated the proportion of mature dendritic cells in the prepared BMDCs. If the proportion of MHC-II-positive cells was higher than 65%, the cells could be used in subsequent experiments (Figure 3c). The phenotypic characteristics of mature DCs included increased expression levels of MHC-II and co-stimulatory molecules. CD80 and CD86 selected in this experiment were co-stimulatory molecules and surface markers of mature DCs. The results showed that the expression levels of CD80 and CD86 significantly increased in all other experimental groups, except for the CD86 level in the mH3HA group (Figure 3d), indicating that LNP/mRNA could stimulate the maturity of dendritic cells. CD80 levels in the mH3N2 group were significantly higher than those in the nuclease-free water group (Mock), which might be due to the ability of mRNA to activate TLRs.

LNPs/mLuc encoding firefly luciferase was inoculated into mice through intranasal and intramuscular administration (right leg) to evaluate the efficiency of the LNPs as a gene delivery vector. The images showed obvious fluorescence in the lungs (Figure 3e,f) and right legs (Figure 3g,h). Irrespective of intranasal or intramuscular administration, the fluorescence intensity in the LNP-Man/mLuc group was higher than that in the LNPs/mLuc group. The addition of mannose ligands might have improved the gene delivery efficiency in vivo.

### 3.4. Construction and Verification of mRNA Vaccine Encoding the HA Protein of A/Jilin/JYT-01/2018(H1N1) Virus 

**Figure 4:**
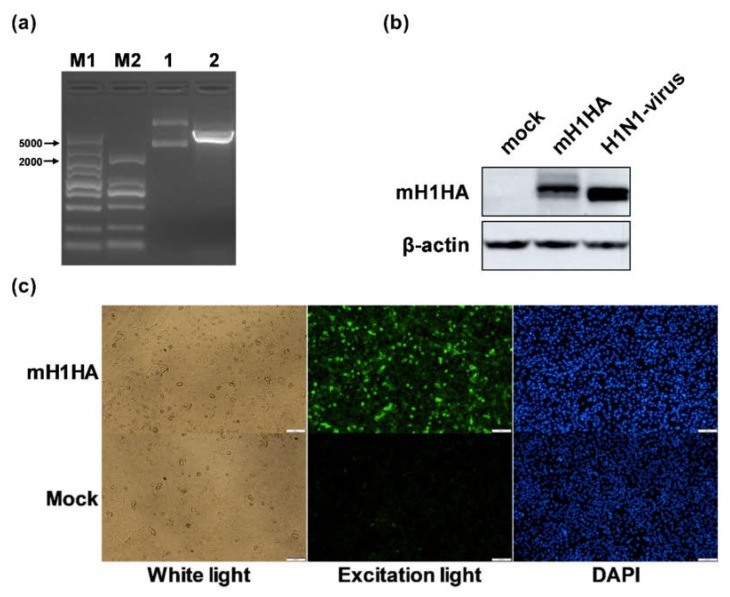
Construction and verification of mRNA vaccine encoding the H1N1-HA protein. (**a**) Agarose gel electrophoresis of *Xho*I enzyme digestion products. 1 represents the intact plasmids of pGEM-H1N1-HA-n3 and 2 represents its linearized product of 5355 bp. M1: DL 5,000 DNA Marker (TaKaRa, Tokyo, Japan); M2: DL 2000 DNA Marker (TaKaRa, Tokyo, Japan). (**b,c**) Western blot and indirect immunofluorescence analyses. A549 cells were harvested 12 h and 48 h after transfection. The H1N1-HA protein was detected using rabbit anti-influenza A virus HA Mab. The H1N1-virus group was used as the positive control. Mock represents the negative control. DAPI was used to dye the nuclei.

Recombinant plasmids encoding the whole length of the HA gene of A/Jilin/JYT-01/2018(H1N1) virus were obtained and verified by *Xho*I restriction enzyme digestion and sequencing (Figure 4a). These results were consistent with the predicted length after adding the UTRs and poly (A) to the vector bone. A549 cells were transfected with mH1HA-n3, and the expression of H1N1-HA protein was confirmed by Western blot analysis (Figure 4b) and indirect immunofluorescence (Figure 4c). These results showed that the HA protein measuring about 75 kDa was successfully expressed. Under excitation light, the green fluorescence could only be observed in the mH1HA transfection group.

### 3.5. Immunization Route and LNPs/mH1HA Vaccines-Induced Functional Antibody Responses in Mice

**Figure 5:**
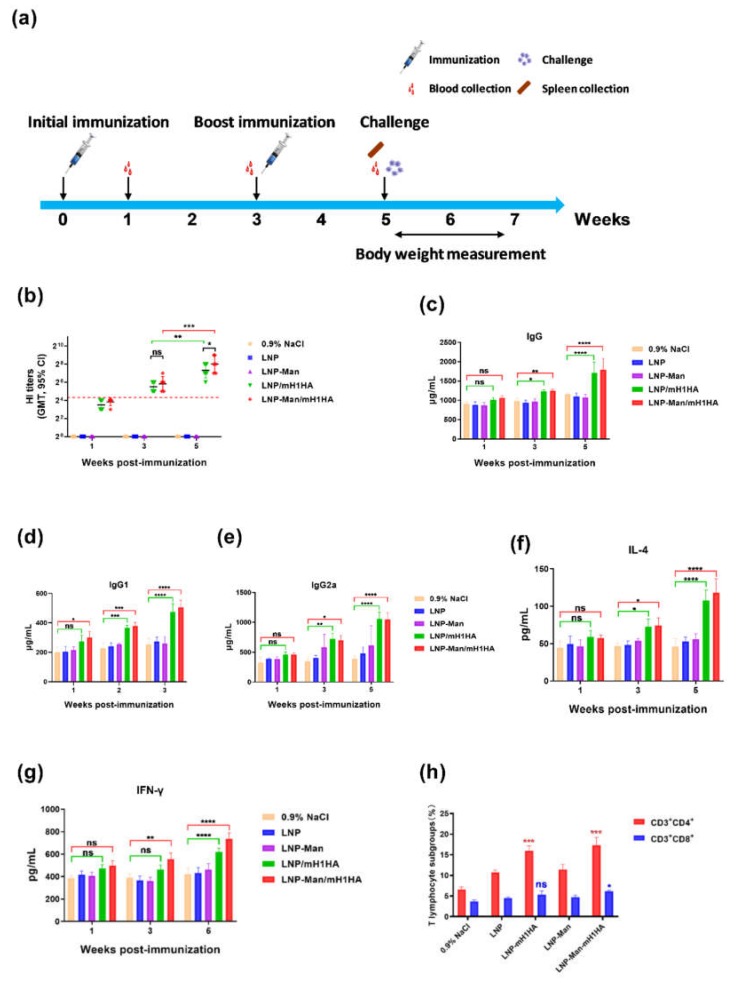
Immune responses in mice induced by LNPs/mH1HA vaccines. Serum samples were collected in the first, third, and fifth weeks after the initial immunization. (**a**) The flow chart of the animal experiment. (**b**) Hemagglutination inhibition (HI) assay. Data are shown as the geometric mean with 95% CI and were analyzed by two-way ANOVA (*n* = 6, ns *p* > 0.05; ** *p* < 0.01; *** *p* < 0.001). (**c–e**) Total IgG/IgG1/IgG2a levels in the serum were detected using ELISA kits. Data are shown as means ± SDs and were analyzed by two-way ANOVA (*n* = 3, ns *p* > 0.05; * *p* < 0.05; ** *p* < 0.01; *** *p* < 0.001; **** *p* < 0.0001). (**f,g**) The IL-4 and IFN-γ levels in sera were determined by ELISA. Data are shown as means ± SDs and were analyzed by two-way ANOVA (*n* = 3, ns *p* > 0.05; ** *p* < 0.01; **** *p* < 0.0001). (**h**) The percentages of CD3^+^CD4^+^ and CD3^+^CD8^+^ T lymphocyte subgroups were tested by flow cytometry. Data are shown as means ± SDs and were analyzed by two-way ANOVA (*n* = 3, ^ns^ *p* > 0.05; * *p* < 0.05; *** *p* < 0.0001, compared with the 0.9% NaCl group).

The methods of vaccination of anti-influenza virus RNA vaccine mainly include intramuscular injection, intradermal injection, subcutaneous injection, and intranasal administration, of which, the intramuscular injection method is the most commonly used. However, we consider that the influenza viruses mainly infect the respiratory system of the human body. If the vaccine can produce effective immune memory in the nasal cavity or lungs, the human body will quickly generate immune responses when the influenza virus invades. At the same time, intranasal administration is more acceptable than other immune routes, especially for children. Therefore, we chose intranasal administration (Figure 5a).

The results of the HI assay represented the levels of anti-H1HA-specific antibody. The HI titers were all more than the protection limit until the third week (1:20, in mice). After boosting immunization, the HI titers of LNPs/mH1HA groups were significantly enhanced; especially, the levels in the LNP-Man/mH1HA group were significantly higher than that in the LNPs/mH1HA group. The serum from mice immunized with 0.9% NaCl, LNP, and LNP-Man was determined to be seronegative (Figure 5b). The total IgG/IgG1/IgG2a levels in the serum from the LNPs/mRNA group were all significantly higher than the levels in the serum from the 0.9% NaCl group in the third and fifth weeks (Figure 5c–e). These results demonstrated that LNPs/mH1HA vaccines could induce a potent antibody response, especially after boosting immunization. The polarization of the T helper (Th) profile was reflected in the IgG2a/IgG1 isotype ratio, where LNPs/mH1HA strongly induced IgG2a subclasses. Therefore, LNPs/mH1HA might have shifted the Th cell profile mainly toward a Th0/Th1 phenotype.

### 3.6. LNPs/mH1HA Vaccines-Enhanced Cytokine Production

Cytokine production is an important indicator for evaluating the immune response. Overall, the cytokine levels in the fifth week were higher than those in the third week. The IL-4 and IFN-γ levels in the LNPs/mRNA group were significantly higher than those in the 0.9% NaCl group in the fifth week (Figure 5f,g). These results demonstrated that LNPs/mH1HA vaccines could enhance the production of both Th1-associated IFN-γ and Th2-associated IL-4.

### 3.7. LNPs/mH1HA Vaccines Induced Functional T-cell Responses in Mice

Two weeks after the boosting immunization, the splenocytes were isolated from three mice of each group and incubated simultaneously with an APC anti-mouse CD3ε antibody and an FITC anti-mouse CD4 antibody (or PE anti-mouse CD8a antibody) (BioLegend, San Diego, CA, USA). The flow cytometric results showed that the LNPs/mH1HA vaccines could significantly enhance the responses of CD4 T-cell, but the CD8 T-cell responses were observed only in the LNP-Man/mH1HA group (Figure 5h). The vaccine might mainly induce the CD4 subtype T-cell responses.

### 3.8. Protection of LNPs/mH1HA From 10×LD_50_ H1N1 Influenza Virus Challenge in Mice

**Figure 6:**
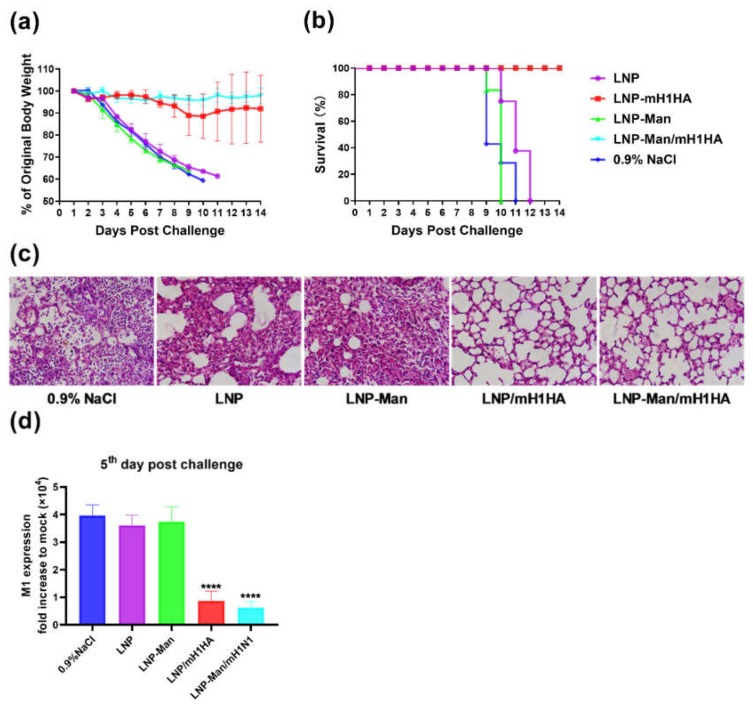
Evaluation of the protective effects after the challenge. (**a**) Body weight changes and (**b**) Kaplan–Meier survival curves of H1N1-infected C57BL/6 mice (*n* = 6) treated with vaccines or vehicles by intranasal administration. Two weeks after the boost immunization, mice were infected with 10 × LD_50_ A/Jilin/JYT-01/2018(H1N1) influenza virus. Lungs were collected at 5th day post-infection. (**c**) Representative images of lung pathology in hematoxylin-and-eosin-stained sections from H1N1-infected C57BL/6 mice (*n* = 3). (**d**) Viral loads expressed as the fold change compared to noninfected lungs. Data are shown as means ± SDs and were analyzed by one-way ANOVA (*n* = 3, **** *p* < 0.0001, compared to 0.9% NaCl, LNP, and LNP-Man groups).

The median lethal dose of the H1N1 influenza virus was previously determined by the Reed and Muench method (LD_50_ = 10^-3.625^/0.1 mL). Two weeks after the boosting immunization, the mice in the five groups were challenged intranasally with 10× LD_50_ H1N1 influenza virus. The animals were monitored daily for body weight (Figure 6a) and survival rate (Figure 6b) for two weeks (six mice/group). The results showed that immunization with LNPs/mH1HA and LNP-Man/mH1HA protected mice from weight loss and death. In contrast, mice immunized with OVA, LNP, or LNP-Man all died within 12 days after the challenge.

The images of lung pathology (Figure 6c) showed that the number of infiltrating cells in mouse lung tissues significantly decreased in the vaccination groups (LNPs/mH1HA and LNP-Man/mH1HA), and the outline of the alveoli was clear. On the contrary, the results of the control groups showed that the infiltrating cells in the alveolar cavity increased (0.9% NaCl), the alveolar wall thickened (LNP), and most of the alveolar contours disappeared (LNP-Man). Figure 6d showed that the lung virus loads in the vaccine groups were significantly lower than those in the control groups on the fifth day after challenge. We considered that the LNP/mH1HA and LNP-Man/mH1HA could effectively inhibit the replication of the H1N1 influenza virus in lung tissue of mice.

## 4. Discussion

This study aimed to develop mRNA vaccines against the H1N1 influenza virus infection. Various in vitro transcription systems and nonviral delivery vectors were selected for mRNA vaccine design. To verify the effect of 5’ and 3’ UTR sequences on translation efficiency of mRNA, three modes of IVT-mRNA-systems (IVT-mRNA-n1/n2/n3) with diverse UTRs were constructed, and EGFP and H3N2-HA genes were introduced into each of them. The results showed that after transfection with the same dose of (Lipo3000/mEGFP)-n1/-n2/-n3, the percentage of EGFP-positive cells in the mEGFP-n3 group was significantly higher than those in the other two groups. Additionally, after transfection with the same dose of (Lipo3000/mH3N2-HA)-n1/-n2/-n3, the results of Western blot analysis showed that the protein expression in the mH3HA-n3 group was the highest. Based on these results, we considered that UTRs in IVT-mRNA-n3 could significantly enhance the translation efficiency of intracellular mRNA, as compared with the other two groups. To further verify the effect of the IVT-mRNA-n3 system, six kinds of cells were transfected with mEGFP-n3, including BHK-21, MDCK, HeLa, HEK-293, BEAS-2B, and NCI-H226. Fluorescence microscope images showed that EGFP could be well expressed in all cell lines. Therefore, IVT-mRNA-n3 was selected for further experiments.

Cationic lipid/mRNA nanoparticles can protect mRNA from degradation by nucleases and deliver it into cells by electrostatic adsorption and fusion with the cell membrane. Lipophilic molecules equipped with ligands can specifically target receptors on the cell surface and improve delivery efficiency. Two kinds of liposomes, LNP and LNP-Man, were prepared to verify whether the gene delivery efficiency could be improved after adding ligands. The results indicated that both could successfully deliver mEGFP into A549 cells, stimulate the maturation of dendritic cells, and promote the expression of mLuc (luciferase) at the inoculation sites in vivo. Overall, the protein expression in the LNP-Man/mRNA group was better than that in the LNPs/mRNA group both in vitro and in vivo.

Therefore, an IVT-mRNA-n3 system and two kinds of liposomes were used to prepare specific mRNA vaccines against the H1N1 influenza virus.

The data showed that the LNPs/mH1HA vaccines could induce HA-specific immune responses (HI assay); generate antibodies (IgG, IgG1, and IgG2a); produce cytokines (IL-4 and IFN-γ); and induce T-cell responses (mainly CD4 T lymphocytes) in mice. The levels of HI titers, IgG/IgG1/IgG2a, and cytokines significantly increased following boosting immunization. High levels of HI titers illustrated that specific antibodies against the H1N1-HA protein were produced. The polarization of the T helper (Th) profile was reflected in the IgG2a/IgG1 isotype ratio, where LNPs/mH1HA strongly induced IgG2a subclasses. Therefore, LNPs/mH1HA might have mainly shifted the Th cell profile toward a Th0/Th1 phenotype. The cytokine production demonstrated that LNPs/mH1HA vaccines could enhance the production of both Th1-associated IFN-γ and Th2-associated IL-4. Overall, the LNPs/mH1HA vaccines could elicit both humoral and cellular immune responses. Two weeks following the boosting immunization, the mice were challenged intranasally with 10× LD_50_ H1N1 influenza virus. The results showed that immunization with LNPs/mH1HA and LNP-Man/mH1HA could protect mice from weight loss and death. The degree of lung injury in the vaccine groups was less than that in the control groups.

## 5. Conclusions

To summarize, 5’ and 3’ UTRs originating from human β-globulin were better than those from human α-globulin in the design of the IVT-mRNA system. Moreover, their sequences and arrangement modes also influenced the translation efficiency of mRNA. LNP equipped with a mannose ligand executed better gene delivery efficiency both in vitro and in vivo. Choosing appropriate ligands might be very helpful in vaccine design. 

This study showed that this LNPs/mRNA platform could be used in the construction of mRNA vaccines. In future studies, this platform may be used to construct a universal anti-influenza virus mRNA vaccine, including the mixed mRNAs of H1N1, H3N2, and influenza B viruses, and evaluate the immune effect in mice infected with homologous and heterologous influenza viruses. This might be helpful in providing valuable experience in the process of vaccine design and experimental verification, thus laying the foundation for the research on the universal influenza virus mRNA vaccine.

In general, many factors affect the efficacy of mRNA vaccines, including optimization of target genes, incorporation of UTRs, selection of delivery vectors, modification of ligands, and addition of vaccine adjuvants. Therefore, researchers can transform and innovate the existing mRNA vaccine platform from multiple aspects and develop a safer, more stable, more convenient, and more effective mRNA vaccine to protect humans from influenza virus infection.

## Supplementary Materials

The following are available online at https://www.mdpi.com/2076-393X/8/1/123/s1, Figure S1. (a) Agarose gel electrophoresis of pGEM-EGFP-n1/n2/n3 digested by *Xho*I enzyme. (b) Agarose gel electrophoresis of pGEM-H3HA-n1/n2/n3 digested by *Xho*I enzyme; Figure S2. The gating strategys of flow cytometry to detect the percentage of EGFP positive cells. (a) The control group. (b) The Cap-mEGFP-n1 group. (c) The Cap-mEGFP-n2 group. (d) The Cap-mEGFP-n3 group; Figure S3. Size distributions of (a) LNP. (b) LNP-Man. (c) LNP/mH3HA. and (d) LNP-Man/mH3HA (N/P=10:1); Table S1. Sequences of tested UTR configurations; Table S2. Mean size (d. nm) of LNPs and LNPs/mRNA. Table S3. Zeta potential (mV) of LNPs and LNPs/mRNA.
