# Supplementary material for: mRNA Vaccines Encoding the HA Protein of Influenza A H1N1 Virus Delivered by Cationic Lipid Nanoparticles Induce Protective Immune Responses in Mice
Source: Vaccines (Basel). 2020 Mar 10;8(1):123. doi: 10.3390/vaccines8010123 (PMC7157730; doi:10.3390/vaccines8010123)
Supplement: Supplementary file 1 [file vaccines-08-00123-s001.pdf]

vaccines

MDPI

Article

mRNA Vaccines Encoding the HA protein of Influenza A H1N1 virus delivered by Cationic Lipid Nanoparticles Induce Protective Immune Responses in Mice

Xinyu Zhuang [1,†], Yanxin Qi [2,†], Maopeng Wang [3], Ning Yu [4], Fulong Nan [5], He Zhang [1], Mingyao Tian [1], Chang Li [1], Huijun Lu [1] and Ningyi Jin [1,*]

1 Key Laboratory of Jilin Province for Zoonosis Prevention and Control, Institute of Military Veterinary Medicine, Academy of Military Medical Sciences, Academy of Military Sciences, Changchun, 130000, Jilin, China; xinyuzhuang367@163.com (X.Z.); hezhangvs@126.com (H.Z.); klwklw@126.com (M.T.); lichang78@163.com (C.L.); huijun_lu@126.com (H.L.)
2 State Key Laboratory of Polymer Physics and Chemistry, Changchun Institute of Applied Chemistry, Chinese Academy of Sciences, Changchun, 130022, China; yxqi@ciac.ac.cn
3 Institute of Virology, Wenzhou University, Wenzhou, 325000, Zhejiang, China; wangmaopenga@126.com
4 Department of Veterinary Medicine, College of Agriculture, Yanbian University, Yanji, 133000, Jilin, China; yn8597@126.com
5 College of Veterinary Medicine, Jilin University, Changchun, 130000, Jilin, China; nflcom@163.com
† The authors contributed equally.
* Correspondence: ningyik@126.com; Tel.: +86-431-86985929

Supplementary Materials

Figure S1. (**a**) Agarose gel electrophoresis of pGEM-EGFP-n1/n2/n3 digested by *Xho*I enzyme. 1, 3 and 5 represent the intact plasmids of pGEM-EGFP-n1/n2/n3; 2, 4, 6 represent their linearized products of 4215bp, 4406bp and 4364bp, respectively. (**b**) Agarose gel electrophoresis of pGEM-H3HA-n1/n2/n3 digested by *Xho*I enzyme. 1, 3 and 5 represent the intact plasmids of pGEM-H3HA-n1/n2/n3; 2, 4, 6 represent their linearized products of 5206bp, 5397bp and 5355bp, respectively. M1: λ-EcoT14 I digest (TaKaRa, Tokyo, Japan); M2: DL5,000 DNA Marker (TaKaRa, Tokyo, Japan).

Figure S2. The gating strategys of flow cytometry to detect the percentage of EGFP positive cells. (**a**) The control group. (**b**) The Cap-mEGFP-n1 group. (**c**) The Cap-mEGFP-n2 group. (**d**) The Cap-mEGFP-n3 group.

Figure S3. Size distributions of (**a**) LNP. (**b**) LNP-Man. (**c**) LNP/mH3HA. (**d**) LNP-Man/mH3HA (N/P=10:1).

Table S1. Sequences of tested UTR configurations.

Table S2. Mean size (d. nm) of LNPs and LNPs/mRNA.

Table S3. Zeta potential (mV) of LNPs and LNPs/mRNA.

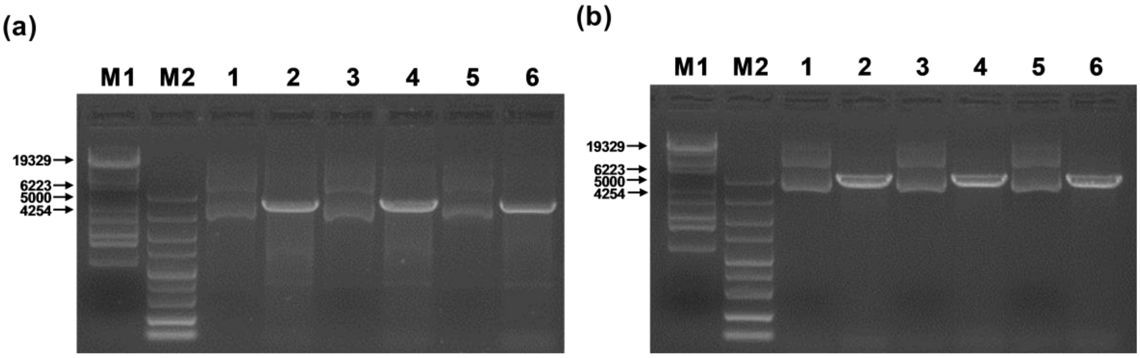

Figure S1. (**a**) Agarose gel electrophoresis of pGEM-EGFP-n1/n2/n3 digested by *Xho*I enzyme. 1, 3 and 5 represent the intact plasmids of pGEM-EGFP-n1/n2/n3; 2, 4, 6 represent their linearized products of 4215bp, 4406bp and 4364bp, respectively. (**b**) Agarose gel electrophoresis of pGEM-H3HA-n1/n2/n3 digested by *Xho*I enzyme. 1, 3 and 5 represent the intact plasmids of pGEM-H3HA-n1/n2/n3; 2, 4, 6 represent their linearized products of 5206bp, 5397bp and 5355bp, respectively. M1: λ-EcoT14 I digest (TaKaRa, Tokyo, Japan); M2: DL5,000 DNA Marker (TaKaRa, Tokyo, Japan).

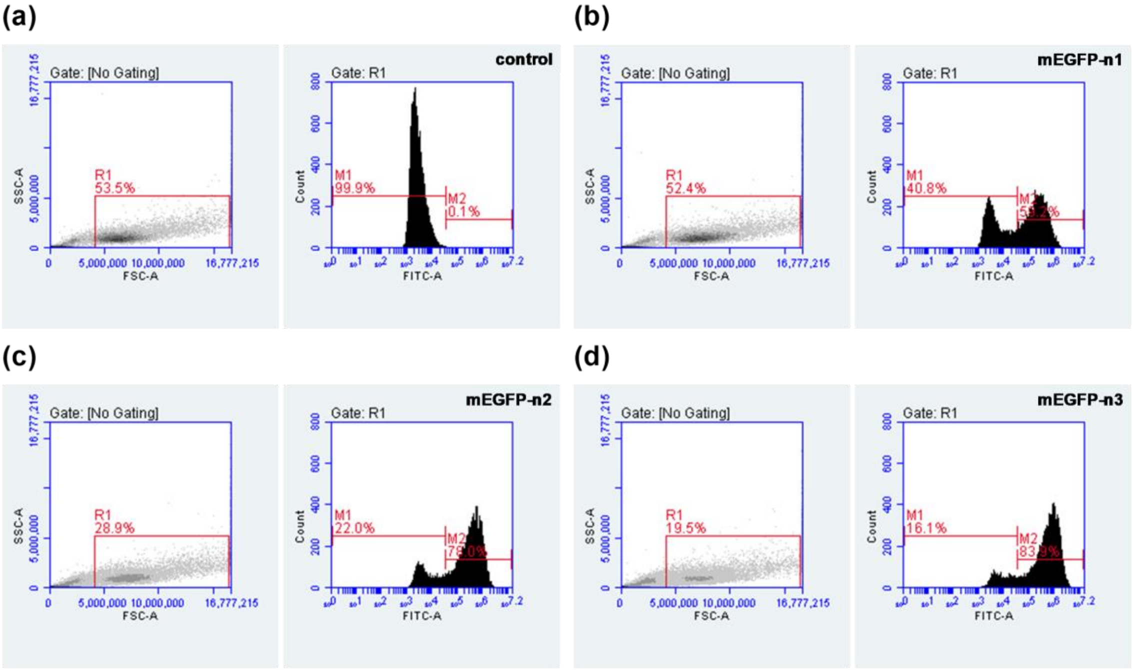

Figure S2. The gating strategys of flow cytometry to detect the percentage of EGFP positive cells. (**a**) The control group. (**b**) The Cap-mEGFP-n1 group. (**c**) The Cap-mEGFP-n2 group. (**d**) The Cap-mEGFP-n3 group.

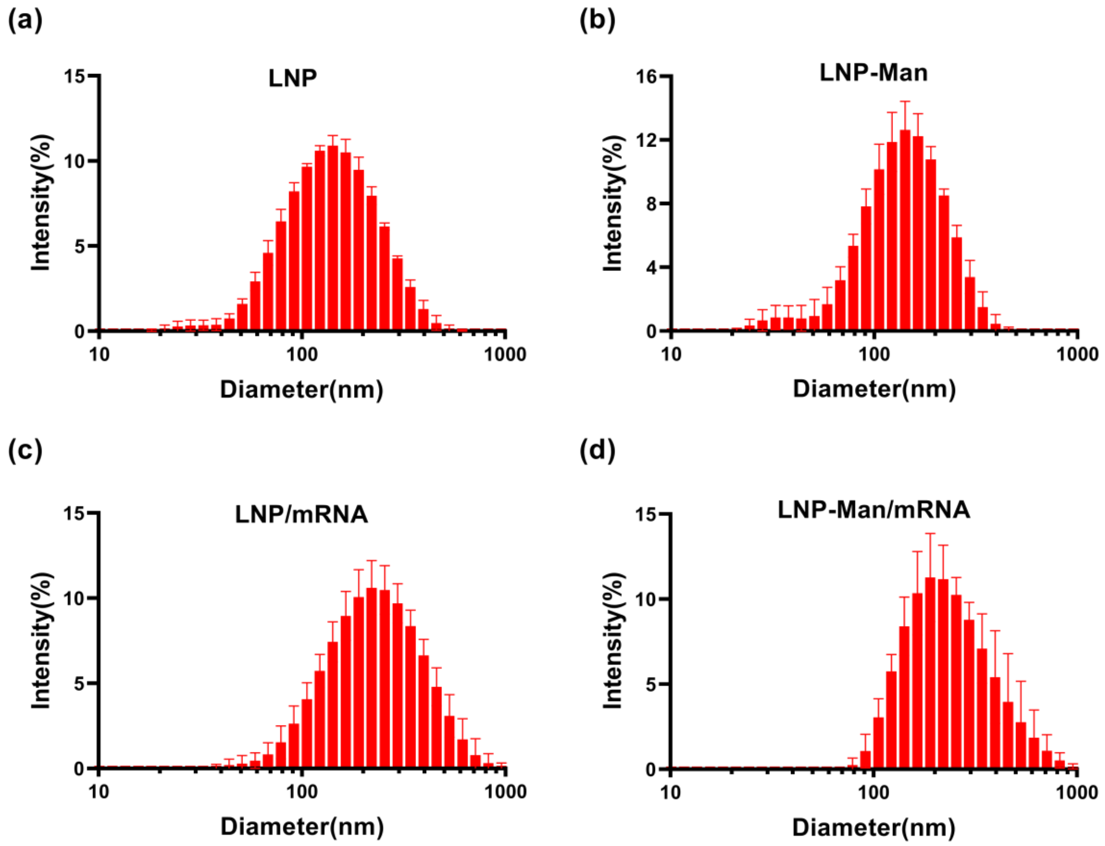

Figure S3. Size distributions of (**a**) LNP. (**b**) LNP-Man. (**c**) LNP/mH3HA. (**d**) LNP-Man/mH3HA (N/P=10:1).

Table S1. Sequences of tested UTR configurations.

IVT-mRNA-n1<br>● 5′UTR (α-globin):<br>AAATAAGAGAGAAAAGAAGAGTAAGAAGAAATATAAGA<br>● 3′UTR (α-globin):<br>GCTGGAGCCTCGGTGGCCATGCTTCTTGCCCCTTGGGCCTCCCCCCAGCCCC TCCTCCCCTTCCTGCACCCGTACCCCCGTGGTCTTTGAATAAAGTCTGAGTG GGCGGC
IVT-mRNA-n2<br>● 5′UTR (β-globin-1):<br>CAGGGCAGAGCCATCTATTGCTTACATTTGCTTCTGACACAACTGTGTTCAC TAGCAACCTCAAACAGACACC<br>● 3′UTR (2β-globin):<br>AGCTCGCTTTCTTGCTGTCCAATTTCTATTAAAGGTTCCTTTGTTCCCTAAGT CCAACTACTAAACTGGGGGATATTATGAAGGGCCTTGAGCATCTGGATTCT GCCTAATAAAAAACATTTATTTTCATTGCAGCTCGCTTTCTTGCTGTCCAATT TCTATTAAAGGTTCCTTTGTTCCCTAAGTCCAACTACTAAACTGGGGGATAT TATGAAGGGCCTTGAGCATCTGGATTCTGCCTAATAAAAAACATTTATTTTC ATTGC

IVT-mRNA-n3

- 5′UTR (β-globin-2):

AGAGCGGCCGCTTTTTCAGCAAGATTAAGCCCAGGGCAGAGCCATCTATTG
CTTACATTTGCTTCTGACACAACTGTGTTCACTAGCAACCTCAAACAGACA
CC

- 3′UTR (2β-globin):

AGCTCGCTTTCTTGCTGTCCAATTTCTATTAAAGGTTCCTTTGTTCCCTAAGT
CCAACTACTAAACTGGGGGATATTATGAAGGGCCTTGAGCATCTGGATTCT
GCCTAATAAAAAACATTTATTTTCATTGCAGCTCGCTTTCTTGCTGTCCAATT
TCTATTAAAGGTTCCTTTGTTCCCTAAGTCCAACTACTAAACTGGGGGATAT
TATGAAGGGCCTTGAGCATCTGGATTCTGCCTAATAAAAAACATTTATTTTC
ATTGC

Table S2. Mean size (d. nm) of LNPs and LNPs/mRNA.

Materials	—	+ mRNA	Change folders
LNP	119.8	203.5	1.70 ↑
LNP-Man	118.3	231.5	1.96 ↑

Table S3. Zeta potential (mV) of LNPs and LNPs/mRNA.

Materials	—	+ mRNA	Change folders
LNP	35.5	18.3	0.52 ↓
LNP-Man	39.3	18.4	0.47 ↓
